# Human chorionic gonadotropin and its relation to grade, stage and patient survival in ovarian cancer

**DOI:** 10.1186/1471-2407-12-2

**Published:** 2012-01-03

**Authors:** Miriam Lenhard, Alexandra Tsvilina, Lan Schumacher, Markus Kupka, Nina Ditsch, Doris Mayr, Klaus Friese, Udo Jeschke

**Affiliations:** 1 Department of Obstetrics and Gynecology, Grosshadern Campus, Ludwig-Maximilians-University Hospital, Marchioninistrasse 15, 81377 Munich, Germany; 2 Department of Obstetrics and Gynecology, Campus Innenstadt, Ludwig-Maximilians-University Hospital, Maistrasse 11, 80337 Munich, Germany; 3 Department of Pathology, Ludwig-Maximilians-University Hospital, Thalkirchner Str. 36, 80337 Munich, Germany

**Keywords:** hCG, LH receptor, Ovarian cancer, Prognosis

## Abstract

**Background:**

An influence of gonadotropins (hCG) on the development of ovarian cancer has been discussed. Therefore, we quantified serum hCG levels in patients with benign and malignant ovarian tumors and the hCG expression in ovarian cancer tissue in order to analyze its relation to grade, stage, gonadotropin receptor (LH-R, FSH-R) expression and survival in ovarian cancer patients.

**Methods:**

Patients diagnosed and treated for ovarian tumors from 1990 to 2002 were included. Patient characteristics, histology including histological subtype, tumor stage, grading and follow-up data were available. Serum hCG concentration measurement was performed with ELISA technology, hCG tissue expression determined by immunohistochemistry.

**Results:**

HCG-positive sera were found in 26.7% of patients with benign and 67% of patients with malignant ovarian tumors. In addition, significantly higher hCG serum concentrations were observed in patients with malignant compared to benign ovarian tumors (*p *= 0.000). Ovarian cancer tissue was positive for hCG expression in 68%. We identified significant differences in hCG tissue expression related to tumor grade (*p *= 0.022) but no differences with regard to the histological subtype. In addition, mucinous ovarian carcinomas showed a significantly increased hCG expression at FIGO stage III compared to stage I (*p *= 0.018). We also found a positive correlation of hCG expression to LH-R expression, but not to FSH-R expression. There was no significant correlation between tissue hCG expression and overall ovarian cancer patient survival, but subgroup analysis revealed an increased 5-year survival in LH-R positive/FSH-R negative and hCG positive tumors (hCG positive 75.0% vs. hCG negative 50.5%).

**Conclusions:**

Serum human gonadotropin levels differ in patients with benign and malignant ovarian tumors. HCG is often expressed in ovarian cancer tissue with a certain variable relation to grade and stage. HCG expression correlates with LH-R expression in ovarian cancer tissue, which has previously been shown to be of prognostic value. Both, the hormone and its receptor, may therefore serve as targets for new cancer therapies.

## Background

Due to missing early clinical symptoms, ovarian cancer is often diagnosed at an advanced stage [1]. Primary treatment includes operative cytoreduction and subsequent combined platinum-based chemotherapy. Though reported primary response rates range around 80%, ovarian cancer is the most lethal gynecological malignancy since 60-70% of patients relapse or die within 5 years after primary diagnosis [2-4].

The molecular mechanism of ovarian cancer development is still discussed controversially [5]. As ovaries are the target organs of gonadotropins, a relation to the development or growth of ovarian cancer has been postulated [6]. An increased risk for the development of ovarian cancer was assumed in women treated for infertility who had therefore been stimulated with gonadotropins [7-9].

Human gonadotropin (hCG) is expressed in placental trophoblasts, but also in a large number of tumors. HCG and the gonadotropin luteal hormone (LH) bind to the same receptor (LH-R) and have similar biological functions, although hCG is more potent because of its higher receptor binding affinity and its longer circulatory half life. Human chorionic gonadotropin is a glycoprotein produced by the fetal trophoblast during pregnancy and is secreted into the maternal circulation [10]. The commitment of cytotrophoblasts to syncytiotrophoblasts is associated with activation of α- and β-hCG subunit genes [11]. These intermediates are transient, they differentiate to syncytiotrophoblasts and the expression of β-hCG RNA declines [12]. Also in chorion carcinoma cells consisting of clusters of cytotrophoblast-like and large multinucleated cells, α- and β-hCG RNA is expressed [13]. In these cells, hCG has been used as a tumor marker for a long time [14].

There are only few studies with small patient numbers on human chorionic gonadotropin and its receptor expression in ovarian cancer tissue [15,16]. In a previous study we found a prognostic value of LH-R and FSH-R in ovarian cancer patients [17]. The present study was designed to further analyze hCG expression in a large cohort of ovarian cancer patients and its relation to histological subtype, grade, stage, gonadotropin receptor expression and patient survival. In addition, we determined hCG serum concentrations in patients with ovarian cancer and compared the results to patients with benign ovarian tumors.

## Methods

### Sera

Sera of patients diagnosed with an ovarian tumor between 2003 and 2006 were obtained before surgery and stored at -80°C. After surgery, histological diagnostic evaluation including staging and grading of tumor tissue were performed by an experienced gynecologic pathologist (D.M.) according to the criteria of the International Federation of Gynaecologists and Obstetricians (FIGO) and the World Health Organization (WHO).

### Tissue samples

All tissue samples were gained at surgery in patients who had been treated for primary ovarian cancer at our institution between 1990 and 2002. Staging and grading were performed by an experienced gynecologic pathologist according to the criteria of the International Federation of Gynaecologists and Obstetricians (FIGO) and the World Health Organization (WHO). Patients with ovarian borderline tumor were excluded from the study. Clinical data of the patients' disease were available from patient charts, aftercare files and tumor registry database information. The main outcomes assessed were disease recurrence and patient survival.

### Ethics approval

The study has been approved by the local ethics committee of the Ludwig-Maximilians University Munich (approval with the reference number 138/03) and has been carried out in compliance with the guidelines of the Helsinki Declaration of 1975. The study participants gave their written informed consent and samples and clinical information were used anonymously.

### hCG-ELISA

Concentration of hCG was obtained by an ELISA and using the Immulite 2000 automated diagnostic system (Siemens, Munich, Germany). Standard deviation for precision at 6.5 m IU/ml is 0.43 with a variation coefficient (CV) of 6.6%. Precision analysis showed no cross reactivity with human FSH (26.8 ng/ml analyzed), LH (16.5 ng/ml analyzed) or TSH (860 ng/ml analyzed).

Immunohistochemistry was performed as previously described elsewhere, using a combination of pressure cooker heating and the standard streptavidin-biotin-peroxidase complex with the use of the rabbit-IgG-Vectastain Elite ABC kit (Vector Laboratories, Burlingame, CA) [18,19]. Antibodies used for staining were the anti-hCG (17.75 μg/ml, rabbit IgG, polyclonal, dilution 1:400, Dako, Glostrup, Denmark) and anti-LH (LH/hCG-R, 1 mg/ml, rabbit IgG, polyclonal, dilution 1:25, Dianova, Hamburg, Germany).

In short, paraffin-fixed tissue sections were dewaxed with xylol for 15 min and then dehydrated in ascending concentrations of alcohol (70%, 96%, and 100%). Afterwards, they were exposed for epitope retrieval for 10 min in a pressure cooker using sodium citrate buffer (pH 6.0) containing 0.1 M citric acid and 0.1 M sodium citrate in distilled water. After cooling, slides were washed in PBS twice. Endogenous peroxidase activity was quenched by dipping in 3% hydrogen peroxide (Merck, Darmstadt, Germany) in methanol for 20 min. Non-specific binding of the primary antibodies was blocked by incubating the sections with "diluted normal serum" (10 ml PBS containing 150 μl horse serum; Vector Laboratories, CA) for 20 min at room temperature. Then, slides were incubated with the primary antibodies at room temperature for 60 min. After washing with PBS, slides were incubated in "diluted biotinylated serum" (10 ml PBS containing 50 μl horse serum; Vector Laboratories, CA) for 30 min at room temperature. After incubation with the avidin-biotin-peroxidase complex (diluted in 10 ml PBS, Vector Laboratories, CA) for 30 min and repeated PBS washing, visualization was conducted using substrate and chromagen 3,3'-diaminobenzidine (DAB; Dako, Glostrup, Denmark) for 8-10 min. Slides were then counterstained with Mayer's acidic hematoxylin and dehydrated in ascending concentrations of alcohol (50-98%). After xylol treatment, slides were covered.

Placental tissue at 3rd trimenon served as a positive control for the hCG and LH-R staining, accordingly. For negative controls, primary antibody was replaced with normal control serum rabbit IgG (BioGenex, San Ramon, USA). Positive staining resulted in brownish color, negative controls as well as unstained cells in blue color.

### Immunohistochemical analysis

Slides were evaluated and digitalized with a Zeiss photomicroscope (Axiophot, Axiocam, Zeiss, Jena, Germany). Immunohistochemical staining was assessed using a semiquantitative score according to Remmele and Stegner [20], comprising optical staining intensity (graded as 0 = no, 1 = weak, 2 = moderate, and 3 = strong staining) and the percentage of positively stained cells (0 = no, 1 = < 10%, 2 = 11-50%, 3 = 51-80% and 4 = > 81% cells). According to previously published data, we scored the tumor tissue as positive if more than 10% of cells were scored with an immunoreactive score (IRS) higher than 2 [15]. The slides were reviewed in a blinded fashion by two independent observers, including a gynecological pathologist (D.M.).

### Statistical analysis

Statistical analysis was performed using SPSS 18.0 (PASW Statistic, SPSS Inc., IBM, Chicago, IL). Correlation analysis of the receptor expression was performed for the histological subtype, tumor stage, grading and clinical data using the non-parametric Kruskal-Wallis rank-sum test and the non-parametric Spearman correlation coefficient. For the comparison of survival times, Kaplan-Meier curves were drawn. The chi-square statistic of the log-rank test was calculated to test differences between survival curves for significance. P values below 0.05 were considered statistically significant.

## Results

### Patient characteristics

Sera of 123 patients diagnosed with either benign (n = 83) or malignant (n = 40) ovarian tumors were obtained before surgery to test for serum hCG levels. Among the patients with benign ovarian tumors were cystadenomas (n = 12), simple ovarian cysts (n = 25), endometriosis (n = 9), teratomas (n = 10), fibromas (n = 8) and other tumors (n = 18). Patients with ovarian carcinomas mostly presented at stage III or IV (FIGO I: 15.4%, FIGO II: 11.5%, FIGO III: 53.8% and FIGO IV: 19.2%). Patients with borderline tumors of the ovary are neither included in the benign nor in the ovarian cancer patient group.

Paraffin embedded tissue of 156 ovarian cancer patients was available. Median age at primary diagnosis was 58 years (range 18-88). Most patients presented with progressed disease at primary diagnosis [FIGO I: n = 35 (22.6%), FIGO II: n = 9 (5.8%), FIGO III: n = 109 (70.3%), FIGO IV: n = 2 (1.3%)]. Patient characteristics are detailed in Table 1. Median follow-up time was 7.3 years (range 0.3-16.8) with 26 documented relapses and 91 deaths. Median relapse free survival was 2.1 years (range 0.9-7.2) and median overall survival 5.9 years (range 0.3-16.6) (Table 1).

**Table 1:** Patient characteristics of ovarian cancer patients whose tissue samples were stained by immunohistochemistry for hCG expression or serum samples were analyzed for hCG concentration

		Tissue samples	Serum samples
**Ovarian cancer patients (n)**		156	40

**Age at primary diagnosis (a)**		58 (range 18-88)	62 (range 21-80)

**Histology (%)**	serous	70.5	81.0
	
	mucinous	13.5	4.8
	
	endometrioid	7.7	14.3
	
	clear cell	8.3	0.0

**Tumor grading (%)**	low grade	27.2	4.2
	
	intermediate	36.5	54.2
	
	high grade	36.3	51.7

**Tumor stage (FIGO) (%)**	I	22.6	13.9
	
	II	5.8	13.9
	
	III	70.3	55.6
	
	IV	1.3	16.7

**Gonadotropin receptor expression (%)**	LH-R positive	64.3	-
	
	FSH-R positive	63.1	-

### hCG ELISA

**Figure 1:**
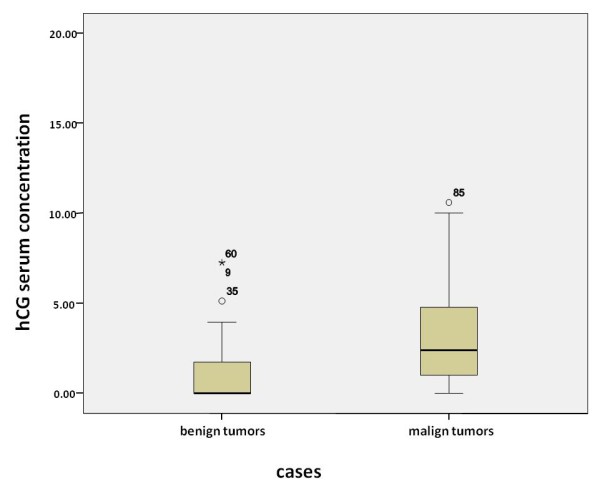
**Determination of hCG in sera of patients with diagnosed benign or malign diseases of the ovaries before surgery shown in boxplots**. The boxes represent the range between the 25th and 75th percentiles with a horizontal line at the median. The bars delineate the 5th and 95th percentiles. The circle indicate values more than 1.5 box lengths, and the asterisk values more than 3.0 box lengths from the 75th percentile. Numbers at circle and asterisk indicate sample number. hCG levels were significantly lower in patients with benign compared to patients with malign diseases of the ovaries (*p *= 0.000).

In serum analysis, we found hCG-positive sera in 26.7% of patients with benign ovarian tumors and 67% positive sera in patients with malignant ovarian tumors. In addition, we identified significant differences in hCG concentration in benign compared to malignant diseases of the ovaries (*p *= 0.000). The median calculation has been done using all samples, i.e. negative samples were also included in the calculation. Median hCG concentration in patient sera with benign ovarian tumors was 0.1 mU/ml and 4 mU/ml in patients with malign ovarian tumors (Figure 1).

### hCG expression in ovarian cancer tissue

**Figure 2:**
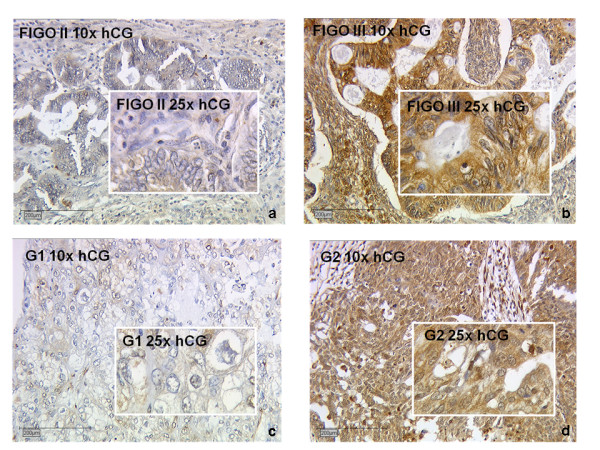
**Representative slides of immunohistochemical staining for hCG expression for FIGO stage II (a, hCG negative), FIGO stage III (b, hCG positive) for grade 1 (c, weak hCG staining) and grade 2 (d, strong hCG staining) ovarian cancer tissue**. No hCG immunoreactivity was detected in tumor stroma (magnification 10× and 25×).

**Figure 3:**
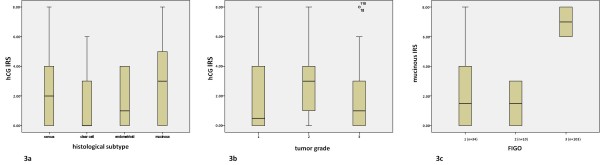
**Expression of hCG in ovarian cancer shown in boxplots**. **a** Expression of hCG in ovarian cancer subtypes shown in boxplots. The boxes represent the range between the 25th and 75th percentiles with a horizontal line at the median. The bars delineate the 5th and 95th percentiles. There were no significant differences in hCG expression between serous, clear cell, endometrioid or mucinous forms of ovarian cancer. **b **Expression of hCG in all ovarian cancer subtypes shown in boxplots regarding to grading. We identified significant differences in G1, G2 and G3 carcinomas (*p *= 0.022). The boxes represent the range between the 25th and 75th percentiles with a horizontal line at the median. The bars delineate the 5th and 95th percentiles. The circle indicates values more than 1.5 box lengths. **c **Expression of hCG in the mucinous ovarian cancer subtype shown in boxplots regarding to staging. We identified significant differences in FIGO I, FIGO II and FIGO III carcinomas (*p *= 0.018). The boxes represent the range between the 25th and 75th percentiles with a horizontal line at the median. The bars delineate the 5th and 95th percentiles.

Immunohistochemical analysis revealed hCG positive tumors in 68% of all cancer tissues investigated (Figure 2a, b). Only slight differences in hCG expression could be observed with respect to the histological subtype, with lowest expression in clear cell carcinomas and highest in mucinous ovarian carcinomas (Figure 3a). Regarding tumor grade, we identified significant differences in hCG expression among G1, G2 and G3 carcinomas (Figure 3b, p = 0.022). With respect to tumor stage, a significant difference was observed in mucinous tumors at stage FIGO I compared FIGO II and FIGO III (*p *= 0.018, Figure 3c).

In addition, a positive correlation of hCG to LH-receptor expression (correlation coefficient 0.194, *p *= 0.037, Table 2) was identified. Interestingly, there was no correlation of hCG expression and FSH-receptor expression.

**Table 2:** Correlation between hCG, LH-receptor and FSH-receptor expression in all ovarian cancer subtypes

Correlations								
			**hCG (Int)**	**hCG (IRS)**	**LH-R (Int)**	**LH-R (IRS)**	**FSH-R (Int)**	**FSH-R (IRS)**

Spearman's rho	hCG (Int)	Correlation Coefficient	1.00	0.94**	0.13	0.19*	-0.09	0.01
		
		Sig. (2-tailed)		< 0.01		0.04		
	
	hCG (IRS)	Correlation Coefficient	0.94**	1.00	0.12	0.19*	-0.01	0.09
		
		Sig. (2-tailed)	< 0.01			**0.04**		
		
		N (Int/IRS)	143	143	116	116	114	113
	
	LH-R (Int)	Correlation Coefficient	0.13	0.12	1.00	0.90**	0.23*	0.23*
		
		Sig. (2-tailed)				< 0.01	0.01	0.01
	
	LH-R (IRS)	Correlation Coefficient	0.19*	0.19*	0.90**	1.00	0.21*	0.24*
		
		Sig. (2-tailed)	0.04	**0.04**	< 0.01		0.03	0.01
		
		N (Int/IRS)	116	116	120	120	116	115
	
	FSH-R (Int)	Correlation Coefficient	-0.09	-0.01	0.23*	0.21*	1.00	0.87**
		
		Sig. (2-tailed)			0.01	0.03		0.01
	
	FSH-R (IRS)	Correlation Coefficient	0.002	0.09	0.23*	0.24*	0.87**	1.00
		
		Sig. (2-tailed)			0.01	0.01	< 0.01	
		
		N (Int/IRS)	113	113	115	115	117	117

We found a significant correlation between hCG and LH-receptor expression whereas hCG expression and FSH-receptor expression do not correlate with each other. (IRS = immunoreactive score, Int = staining intensity)

** Correlation is significant at the 0.01 level (2-tailed)

* Correlation is significant at the 0.05 level (2-tailed)

### Prognostic value of hCG

**Figure 4:**
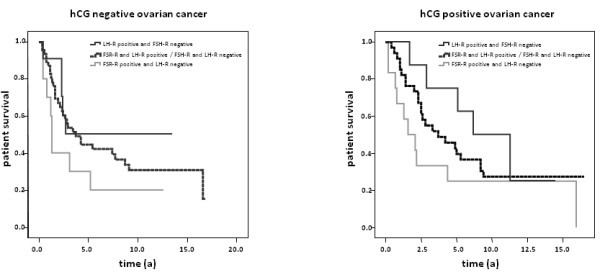
**Kaplan-Meyer analysis indicating survival in subgroups of patients with or without hCG expression in ovarian cancer tissue samples**.

Statistical analysis was performed to test for a prognostic value of hCG expression in ovarian cancer tissue. The univariate Kaplan Meier analysis reveals no statistical difference between patients positive and negative for hCG in ovarian cancer tissue (*p *= 0.618). Interestingly, there was an increased 5-year survival in patients with hCG positive tumors in the LH-R positive/FSH-R negative subgroup (5-year survival: hCG positive 75.0% vs. hCG negative 50.5%; Figure 4, Table 3).

**Table 3:** Ovarian cancer patient survival: 5-year survival for hCG positive and negative tumors with regard to LH receptor (LH-R) and FSH receptor (FSH-R) expression

Ovarian cancer patient 5-year survival (%)	LH-R positive and FSH-R negative	FSR-R and LH-R positive/FSH-R and LH-R negative	FSH-R positive and LH-R negative
hCG negative	50.5	44.9	30.0

hCG positive	75.0	39.8	25.0

## Discussion

To date, the pathogenesis and progression of ovarian cancer remains unclear. There are various hypotheses to explain its etiology, two of them discussing hormonal influence on tumorgenesis [6,21-23]. Some risk factors for the development of ovarian cancer like nulliparity and infertility have been identified in epidemiologic studies [21,24-26]. Ovarian cancer is often diagnosed in postmenopausal women who present with high gonadotropin blood serum levels [27]. Until today, the influence of hormones, especially gonadotropins, on the development or progression of ovarian cancer remains under discussion [23,27,28].

In this study, serum human gonadotropin (hCG) levels differ between patients with benign and malignant ovarian tumors. HCG and its subunits can be measured at low dose in the serum of most men and women [29]. Its values differ according to the level of gonadotropin releasing hormone [30] and it is assumed that most of hCG in serum of healthy persons originates from the pituitary. Studies on hCG-immonoreactivity have demonstrated that hCG is often elevated in serum of patients with gynecological cancers [31]. Still, hCG serum levels seem not to be useful in the diagnosis or therapy monitoring of non-trophoblastic gynecological malignancies [32,33]. But since there is evidence supporting that hCG is produced by gynecological cancers themselves [34-36], hCG production can be suspected to have an influence on gonadotropin receptor expression in cancer tissue. The fact that we observed a positive correlation of hCG to LH-receptor expression supports this assumption.

Among non-trophoblastic cancers, hCG expression is best analyzed in the transitional cell carcinoma of the bladder and urinary tract. The appearance of human chorionic gonadotropin within the tumor cells is described to be an evidence of dedifferentiation, since it is more commonly expressed in poorly differentiated tumors [37]. In this study, we also observed significant differences in hCG expression related to tumor grade (*p *= 0.022) but no differences with regard to the histological subtype. In addition, mucinous ovarian carcinomas showed significantly increased hCG expression at FIGO stage 3 (*p *= 0.018). There is in vitro data with uterine microvascular endothelial cells showing hCG to increase capillary formation and migration of endothelial cells with no effect on cell proliferation [38]. In the same study by Zygmunt et al., hCG was found to induce neovascularization even in ovarian cancer in an in vivo animal model. Therefore, hCG was thought to be an important angiogenetic factor [38]. This finding may in part explain higher hCG expression in dedifferentiated tumors or higher stages in the mucinous ovarian cancer subgroup as observed in our own study. Still, there was no significant difference in patient survival relating to tumor hCG expression as it was found for transitional cell cancer of the bladder [39]. Therefore, we assume hCG to have varying functions in ovarian cancer, e.g. neovascularization or LH-R regulation, which might explain the partly contradictory findings of hCG effects in relation to histological results on the one hand and patient survival on the other.

Interestingly, we found a difference in 5-year survival rate between hCG-positive and hCG-negative tumors depending on LH-R or FSH-R expression. As demonstrated in our previous study, the LH-R and FSH-R themselves have prognostic value for patient's survival [17]. Our results showed a positive correlation of hCG tissue expression and LH-R expression. Therefore we assume hCG also to have an LH-R regulative function. The role of hCG and its receptors in cancer is discussed controversially in literature [15,40]. We have demonstrated here, that the contradictory findings in literature may also be explained by variable gonadotropin hormone and hormone receptor expression.

Gonadotropins bind to extracellular receptors, the LH-R and FSH-R. The LH-R receptor binds not only the gonadotropin LH but also hCG, and is therefore often referred to as LH/hCG-R. It is mainly found in gonadal tissue. Apart from gonadal tissue, it is known to be expressed in a variety of non-gonadal tissues in humans and rodents, like fetal tissues [41], the placenta [42], mammary gland [43], the salpinx, the uterus [44] or the cervix [45]. Most research on this receptor focuses on fertility-related treatments. Nonetheless, new therapeutic fields are evolving regarding this receptor since the LH/hCG-R is expressed in human cancer cells like breast cancer [46] or ovarian cancer [17]. To reduce the side-effects of chemotherapy, receptor-mediated therapies might be a new approach in anticancer treatment. Rahman et al. have developed a lytic peptide, hecate-CGbeta, which selectively kills cancer cells by changing their membrane potential [47]. Its effect on tumor cells has already been proven for breast cancer [46-48] and testicular tumors [49], but also for ovarian cancer [49,50].

Gebauer et al. have analyzed the effect of human chorionic gonadotropin-doxorubicin on ovarian cancer cells and observed an increased activity of doxorubicin when conjugated to hCG [51]. This finding was also described for breast cancer cells [52]. The combination of cytostatic agents with hormones like hCG for the treatment of LH/hCG-R positive cells might be a promising approach to reduce morbidity and mortality in anticancer therapy. New therapeutic agents like lytic peptides or chemotherapeutic agents binding to the LH-R offer less toxic, but effective and selective anticancer treatment options, either alone or in combination with standard chemotherapeutic agents, in ovarian cancer patients whose tumors express these receptors.

Strengths of this study are the long follow-up time, the consistent pathologic histology review by expert gynecologic oncology pathologists and the large sample size. A limitation of this study is obviously the retrospective study design.

## Conclusions

Serum human gonadotropin levels differ in patients with benign and malignant ovarian tumors. HCG is often expressed in ovarian cancer tissue with a certain variable relation to grade and stage. HCG expression correlates with LH-R expression in ovarian cancer tissue, which has previously been shown to be of prognostic value. Both, the hormone and its receptor, may therefore serve as targets for new cancer therapies, which may directly bind to hCG or its receptor LH-R and increase efficacy and specificity of anticancer treatment, thus reducing side effects.

## Competing interests

The authors declare that they have no competing interests.

## Authors' contributions

ML, AT and LS have made substantial contributions to conception, design and acquisition of data. MK, ND and DM have made substantial contributions to analysis and interpretation of data, and have been involved in drafting the manuscript and revising it critically for important intellectual content. KF and UJ have given final approval of the version to be published. In addition, KF and UJ have made substantial contributions to conception and design of the study. All authors read and approved the final manuscript.

## Pre-publication history

The pre-publication history for this paper can be accessed here:

http://www.biomedcentral.com/1471-2407/12/2/prepub
